# Elucidating the Origin of the Attractive Force among Hydrophilic Macroions

**DOI:** 10.1038/srep26595

**Published:** 2016-05-24

**Authors:** Zhuonan Liu, Tianbo Liu, Mesfin Tsige

**Affiliations:** 1 Department of Polymer Science, the University of Akron, Akron, OH 44325, USA

## Abstract

Coarse-grained simulation approach is applied to provide a general understanding of various soluble, hydrophilic macroionic solutions, especially the strong attractions among the like-charged soluble macroions and the consequent spontaneous, reversible formation of blackberry structures with tunable sizes. This model captures essential molecular details of the macroions and their interactions in polar solvents. Results using this model provide consistent conclusions to the experimental observations, from the nature of the attractive force among macroions (counterion-mediated attraction), to the blackberry formation mechanism. The conclusions can be applied to various macroionic solutions from inorganic molecular clusters to dendrimers and biomacromolecules.

Hydrophilic macroions represent a large group of soluble species with sizes from ~1 to 10 nm. Typical macroions include inorganic metal-oxide molecular clusters^1^,^2^,^3^, metal-organic nanocages^4^,^5^, biomacromolecules, dendrimers, and small nanoparticles^6^,^7^ (Fig. 1a). Macroionic solutions cannot be described by either Debye-Hückel theory for simple ions, as the large ions cannot be treated as point charges, or DLVO theory^8^,^9^ for colloids, as the macroions still form real solutions. An important feature of the various macroions is the strong attractive force between like-charged macroions carrying moderate charges, leading to the spontaneous formation of hollow, spherical, single-layered “blackberry” structures in very dilute solutions (thousands of times lower than the maximum solubility of the macroions), with blackberry formation/disassociation and their sizes accurately and reversibly tunable via solvent polarity, macroionic charge density and/or the type of counterions^10^,^11^,^12^,^13^. The blackberry formation is not driven by chemical interaction, hydrophobic interaction or van der Waals forces^14^ (which distinguish macroions from colloids that can be explained by DLVO theory); instead, counterion-mediated attraction is critical^14^,^15^,^16^,^17^. In some cases, hydrogen bonding may also contribute^14^,^15^,^16^. The size disparity between the macroions and their counterions results in significant counterion association around macroions, but this disparity is much less dominant than in colloids^14^,^17^. However, the corresponding complete theoretical explanation is still missing although there exist a few phenomenological models that attempted to capture the nature of self-assembly in these kinds of ions^16^.

The most critical questions to be answered by theory and simulations are the following: (1) what is the source of the attractive force among like-charged soluble macroions with moderate charge density and monovalent counterions, when chemical interaction, hydrophobic interaction and hydrogen bonding are absent? (2) is this attractive force, mediated by counterions, not due to van der Waals forces? and (3) how do the attraction and the assembly size change with macroionic charge density? All these issues have been observed by experiments, but persuasive molecular-level insights into the self-assembly phenomenon of macroionic solutions have hitherto been missing.

Information on the self-assembly of macroions in solution is expected to come primarily from molecular dynamics (MD) simulations. However, very few MD simulations of macroions and their interactions with counterions and other macroions have been reported^18^,^19^,^20^,^21^,^22^,^23^. This is mainly due to the large and complex structures of the macroions, which makes the development of a force field for an all-atom MD simulation of the macroions very difficult. In addition, the assembly of even two macroions in dilute solution using all-atom MD is a very time-consuming simulation. To overcome the difficulties of all-atom MD simulations of macroionic in solution and also to generalize the simulation results (and not be limited to specific kinds of macroions), herein a coarse-grained MD model is introduced in which the atomic details of the macroions are neglected and only the important interactions are emphasized through the van der Waals (VDW) and electrostatic forces (Fig. 1c). In the current model, a macroion is represented by a hollow sphere, mimicking the molecular structure of a most commonly studied 2.5-nm spherical “Keplerate” metal-oxide molecular cluster {Mo_72_Fe_30_}^24^. The wall of the sphere comprises charged and uncharged small beads that are responsible for the electrostatic and VDW interactions of the macroions with the surrounding medium. By using this model, the size of the macroions, the charge density and the surface charge distribution can be easily controlled and, more importantly, simulations with much larger length and time scales become accessible. In the present work, it will be shown that such a coarse-grained model can provide satisfactory answers to some of the questions raised above.

## Results and Discussion

**Figure 2:**
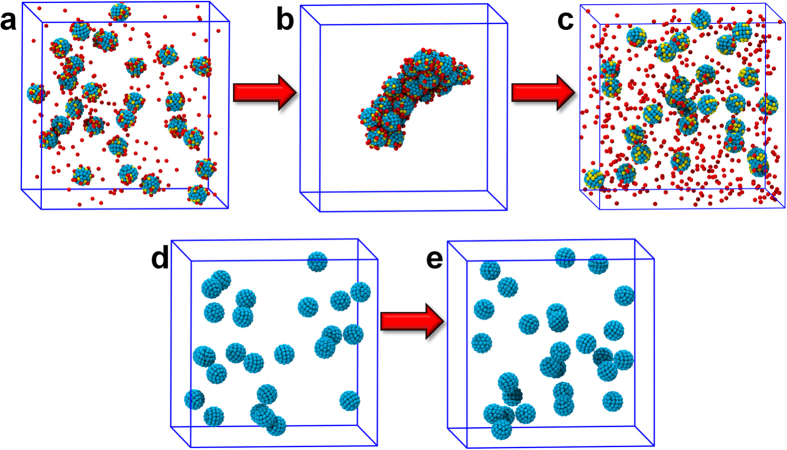
MD simulation snapshots for charged and uncharged macroion in solution. (**a**,**d**) at the beginning and (**b**,**e**) at the end of the simulations, respectively. Coloring: charged beads on macroions are given in yellow, uncharged beads on macroions are cyan, and charged counterions are red. Turning off the charges on the macroions and counterions after aggregate formation and running the simulation further results in a disassembled state (**c**). Solvent beads are not shown for clarity.

To demonstrate whether attractive interactions exist among macroions in solution, 30 charged, 2.5 nm–diameter macroions in solution were simulated. After about 500 ns simulation (more details in the supplementary materials), all the macroions were observed to self-assemble into a single aggregate as shown in Fig. 2b (also see radial distribution function (RDF) shown in Fig. S2b). This clearly shows that the like-charged macroions attract one another to form an aggregate with the counterions distributed among and around them. The counterions are highly mobile, as confirmed by their mean-squared displacement, and from this initial investigation, they seem to be the binding agent that holds the macroions together. The results of this initial simulation demonstrate that the charged macroions do self-assemble in solution despite the fact that they all have identical charges.

The next question regards the nature of the attractive force. In order to clarify this, first, a system similar to the one described above but with no counterions and no charges on the macroions—now referred to as uncharged macromolecules—was simulated. These macromolecules and the macroions in the previous simulation were identical in every respect except charge. This means that, in the current coarse-grained model, the uncharged macromolecules interact with their surrounding environment through van der Waals interactions only. As shown in Fig. 2e, after simulations of more than 500 ns, no sign of any kind of aggregation was observed in the system (RDF shown in Fig. S2a). The macromolecules did come close to each other periodically but never formed even a stable dimer. This implies that the role of van der Waals forces in the self-assembly of macroions in solution should be minimal at best. Intrigued by this observation and in an effort to better understand the role of the van der Waals interactions in the self-assembly process, another simulation was run starting from the aggregated configuration of the charged macroion simulation (Fig. 2b), but with all electrostatic interactions turned off by setting the charges on the macroions and counterions to zero. Within a few picoseconds of simulation, the aggregate disassembled into isolated uncharged macromolecules and “uncharged counterions” in the solution, as shown in Fig. 2c. Put together, these results clearly demonstrate that electrostatic forces, not van der Waals forces, are responsible for the self-assembly of macroions in solution.

**Figure 3:**
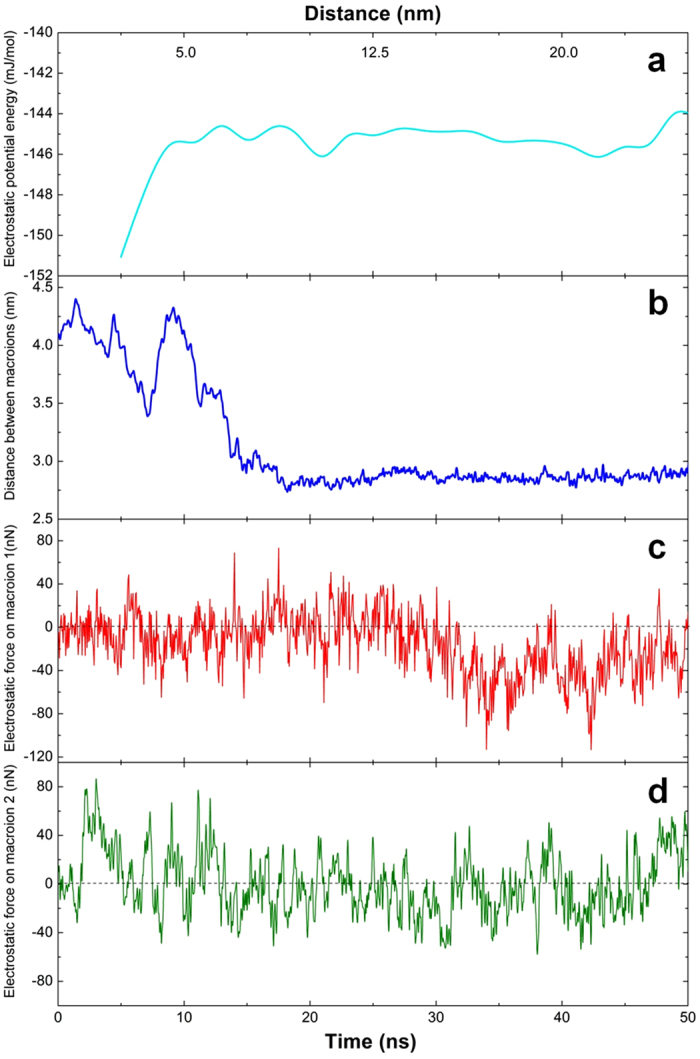
Tracing the energy and force between two macroions in solution. (**a**) The total electrostatic potential energy of the system containing two macroions as a function of the distance between them. For the system containing two macroions, (**b**) is the distance between the two macroions as a function of time as they approach and form a dimer; (**c**,**d**) are the components of the instantaneous total electrostatic force on each macroion in the radial direction between them during dimer formation.

In order to better understand the interactions among macroions, a system containing only two charged macroions in dilute solution was investigated. Due to the low concentration, it took more than 250 ns for the two macroions to form a stable dimer in solution. The average electrostatic energy of the system, shown in Fig. 3a as a function of distance between the two macroions, clearly shows that the dimer is energetically favorable and forms spontaneously–no sign of long-range attraction between the two macroions is observed. Furthermore, the instantaneous distance between the centers of the two macroions, plotted as a function of time in Fig. 3b for the last 50 ns of simulation, also shows no sign of long-range attraction between the two macroions. Similar assembly behavior was observed when the simulation was repeated several times with the macroions randomly placed in the simulation box. There were also a few instances in which the two macroions, before finally forming a stable dimer, came very close to one another (within 3.5 nm) but then departed far away from each other. Taken together, these observations point to essential roles for the counterions in the assembly process, but the specific nature of that role is still elusive.

To that end, the instantaneous total electrostatic force on each macroion in the two-macroion system was calculated, with the projection of this force onto the radial direction between the two macroions monitored as a function of time. Figure 3c,d show the projected instantaneous force for each of the macroions, respectively, during the last 50 ns of simulation. The convention used here is that a negative force is an attractive force exerted on the center of one macroion in the direction towards the center of the other macroion, while a positive force is repulsive. From this, an intriguing picture emerged regarding the roles of the counterions in the self-assembly process. The dimer formation is indeed mediated by counterions, but the process is complex–the rapid change in the sign and magnitude of the forces in Fig. 3c,d is due to the complex dynamics of the counterions around the macroions. A careful examination of the trajectories of the macroions and counterions in the self-assembly process, combined with mean-squared displacement analysis, confirmed that the counterions in between the macroions and around them are diffusing around the macroions and do not form electrical double layer (EDL). However, there is symmetry in this “chaos”, as observed by the periodic oscillation of the forces, which suggests an emerging pattern as the two macroions approach each other. In this situation, some of the counterions start to spend more time in between the two macroions, resulting in an overall attractive electrostatic force between the two macroions and leading to dimer formation. Even after the dimer is formed, the electrostatic forces on the two macroions continue to oscillate in magnitude and direction but no “breaking of the bond” between the macroions was observed. However, through visualization and dynamic analysis, the two macroions were found to rotate and vibrate around the bond, the latter illustrated in Fig. 3b. Furthermore, the magnitude of the average electrostatic force that keeps the two macroions paired is found to be at least two-orders of magnitude larger than the van der Waals force between them (see Fig. S3).

Now it becomes clear that the counterions mediate an effective attractive electrostatic interaction between the macroions that results in self-assembly. But, being very dynamic, they are also the main reason for the slow process of self-assembly, which can take anywhere from several days to several weeks in experiments^25^. The unequivocal conclusion of this work has been confirmed by simulations of a much larger system of 50 macroions and monitoring of the macroion self-assembly in solution. A similar pattern of interaction between the macroions has been observed.

**Figure 4:**
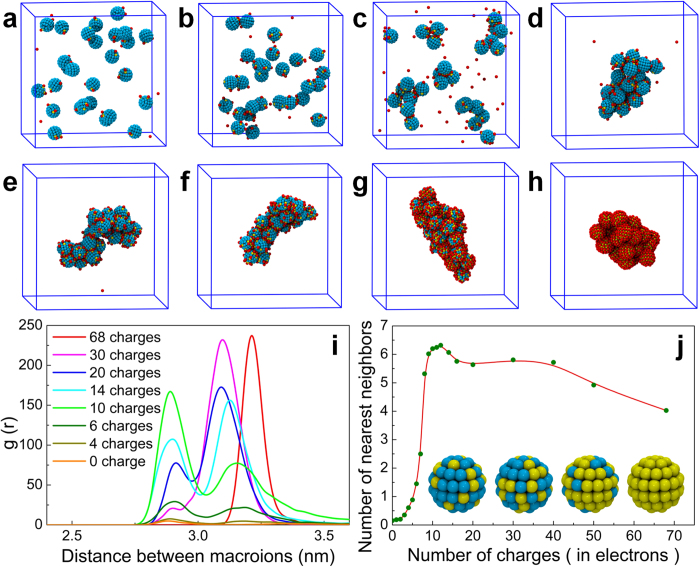
Effect of charge density on the self-assembly of macroions in solution. Self-assembled structures of macroions in solution at equilibrium with different numbers of charged beads on the macroions’ surfaces: 2, 4, 6, 8, 16, 20, 30 and 68 for (**a**–**h**) respectively. The color schemes are the same as in Fig. 1. (**i**) Macroion-macroion radial distribution function, g(r), in the solution for representative numbers of charges on the macroions’ surfaces. (**j**) Number of first nearest neighbors for each macroion as a function of charge on the macroions’ surfaces.

Since the electrostatic forces on the macroions are correlated with the amount of charge on the macroions’ surfaces, how the macroionic surface charge density affects their self-assembly becomes a critical question. To answer this, more than 20 different systems were prepared in which the macroions (2.5 nm in size) had different numbers of charged beads on the surface, varying from 0 to 68 (where all the beads on the macroions are charged). The systems were then simulated until they reached equilibrium, which was defined as observation of no change in the self-assembly behavior for more than 50 ns. The results of these simulations are shown in Fig. 4a–h for eight representative systems. Systems with macroions having less than six charged beads on the surface did not show any sign of big aggregates formation, though small aggregates such as dimers or trimers were observed as the number of charged beads on the surfaces of the macroions increased. Similar small size aggregates were also observed in atomistic MD simulations of the self-assembly of smaller size Keggin anions using Amber force field, in which the counterions were found to be distributed between Keggin anions in order to mediate the formation of small aggregates^22^,^23^. For those charge densities, the electrostatic forces on the macroions were several times larger than the van der Waals forces but were not strong enough to keep several macroions together. With further increase in the charge density, the attractive electrostatic forces among the macroions increased considerably and the macroions started to form larger aggregates with different shapes.

To better understand the aggregated structures, the macroion radial distribution functions for different systems were calculated (Fig. 4i). In general, the radial distribution functions of the different systems show two peaks in close range, the first corresponding to the direct contact between the macroions and the second to the presence of a solvent particle or counterion in between the macroions. The magnitudes and locations of the peaks are strongly dependent on the macroion surface charge density. For low surface charge densities, the first peak is dominant since the low concentration of counterions around the macroions favors direct contact between the macroions. As the surface charge density increases, the first peak decreases while the second peak increases since counterions or solvents have to come in between the macroions to screen the strong repulsive electrostatic force between them. In effect, these two peaks constitute the first nearest neighbor shell, and integrating the area under these peaks for each surface charge density system should give the average number of first nearest neighbors each macroion has in the system. Figure 4j shows the number of first nearest neighbors as a function of the macroion surface charge density, which clearly shows that the transition from no-assembly to self-assembly happens around 5 to 6 charged beads on the surface, and the macroions are loosely self-assembled when the charge density on them becomes too high, due to the repulsive electrostatic forces between macroions and associated counterions becoming dominant. These results are fully supported by experiments^17^.

**Figure 5:**
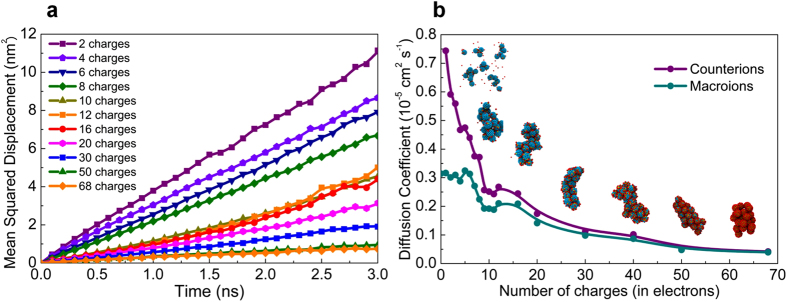
Dynamics of macroions and counterions in solution. (**a**) Mean-squared displacement as a function of time for counterions in different solutions varying with the charge density on macroions. Each curve is a time-average calculation over a single trajectory. For each system the diffusion coefficient was obtained after the aggregated structures were stable for more than 100 ns. (**b**) Comparison of diffusion coefficients of macroions and counterions as a function of number of charges on each macroion. The shapes of the final aggregates in some of the cases are also shown above at the corresponding charge density of the macroions. Error bars are within the size of the symbols.

Furthermore, the dynamics of the different components in the solution were characterized through mean-squared displacement (MSD) calculations. Their self-diffusion coefficients were then determined from a linear fit to the MSD data. However, because of the faster dynamics of CG models due to the smoother interactions compared to atomistic interactions, as explained in MARTINI force field literatures^26^,^27^, a factor of 4, which is the standard conversion factor in MARTINI force filed^26^, was used to rescale the diffusion coefficients of different species in this work. Using this method and also the force field parameters used for our system (see methods section below), the calculated self-diffusion coefficient of solvent is about 2.7 × 10^−5^ cm^2^ s^−1^ at room temperature, which is higher than the bulk diffusion coefficient of bulk water in CG water model in MARTINI (2.0 × 10^−5^ cm^2^ s^−1^) and the experimental measured value of water (2.3 × 10^−5^ cm^2^ s^−1^ at 300 K)^28^. The diffusion coefficient of solvent is the almost same in all the solutions. The dynamics of macroions and counterions were also investigated using the same method, and the results are shown in Fig. 5. Figure 5a shows how the MSD of counterions is changing with increasing of the charge density of macroions. It is clear from the figure that the dynamics of counterions becomes slower as the charge of the macroions is increased. To better understand the correlation of the dynamics between different species in the system, we further calculated the diffusion coefficients of both macroions and counterions as a function of the number of charges on the macroions as shown in Fig. 5b. In general, the dynamics of both counterions and macroions decreases with increase in the surface charge density of macroions, except at low charge densities. At low charge densities, the diffusion coefficient of macroions is constant, within the error of the simulation, since they are not able to form aggregates bigger than trimers while the dynamics of counterions decreases due to their increased association with the macroions. Macroions with more than 5 charges can form bigger aggregates, leading to a drop in the diffusion coefficient of both macroions and counterions due to a dramatic increase in the number of nearest neighbors of macroions (see Fig. 4j). Counterions that are freely moving and also closely associated with macroions have been observed (see Fig. S4) and they switch back-and-forth between the two states. The number of freely moving counterions seems to decrease significantly with increase in the surface charge density.

There is a significant change in the dynamic behavior of both macroions and counterions for more than 10 charges on the macroions surface. Their dynamics are significantly reduced mainly due to more association of macroions and counterions resulting in a higher repulsion between macroions inside the aggregate which consequently enlarge the size of the aggregate. The difference between counterions’ and macroions’ dynamics is always reduced by raising the charge density of macroions. The counterions move with the macroions for more than 30 charges on the macroions surface.

We hypothesize that when macroions have moderate charge densities, they may initially form small 1-D or 2-D aggregates, then after long enough time, through rearrangements of the positions of charged sites on the surface of macroions as well as the counterions, the small aggregates assemble into big monolayers and eventually form a hollow spherical blackberry structure. Mani *et al*. have performed Monte Carlo simulation of macroions without counterions and explicit solvent but with patchy sites on the surface mimicking the hydrogen bonding interactions, and obtained crystal structures of stacks of self-assembled monolayers^29^,^30^. Although those simulations clearly ignored the importance of counterion mediated attractions, which is the main driving force of self-assembled structures formation and also much stronger than the hydrogen bonding, the idea of emphasizing the importance of the positions of interacting sites on macroion’s surface is favorable to our hypothesis of how rearrangement of charged sites and counterions on the surface of macroions would control the blackberry structure formation. Future investigation will be made regarding this hypothesis.

In conclusion, we have used large-scale molecular dynamics simulations with a coarse-grained model specifically designed for macroions in solution to answer some of the most outstanding questions about the solution behavior of macroions; such as the source of the attractive force among like-charged soluble macroions and how the charge density affects the self-assembly behavior. The coarse-grained simulation approach used in the present study offers the potential to observe the macroions’ self-assembly into a blackberry structure using simulations. We believe our general approach of understanding the process of self-assembly of charged molecules in solution will open a new direction in the study of the self-assembly and the nature of interactions in the broadly defined macroionic solutions, which cover a variety of fields from materials science to biological phenomena.

## Methods

### Coarse-grained molecular dynamics simulation

A flexible coarse-grained (CG) model that represents macroions of varying charge and size was developed for the current work. One macroion is represented by one hollow sphere with two different types of beads on the surface. The surface beads are either uncharged or charged in order to represent the van der Waals and electrostatic interactions between macroions and solvent molecules in the solution, as shown in Fig. S1. The size and charge value of each surface bead, the size of the macroion, and the number of charged beads and their distribution on the surface can all be tuned to represent a specific macroion. The surface beads of a macroion are designed to move as one rigid body to avoid the need to define bond and angle terms between them. This is based on the assumption that the shape, size and composition of each macroion will not change in the process of self-assembly in solution, which means that there is no obvious relative movement between atom groups on the surface of each macroion.

The Lennard-Jones (LJ) 12-6 potential energy function was used to describe VDW interactions between the different kinds of beads in the solution


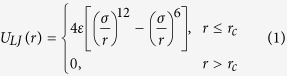


where *U*_*LJ*_(*r*) is the VDW interaction between pairs of beads separated by a distance of *r, ε* is the energy term and *r*_*c*_ is the cut-off distance for LJ potential. The CG force field parameters for solvent were taken from the model of water in MARTINI force field^26^ in which the hydrogen bond interactions between water molecules are also taken into account. The CG force field parameters of the solvent can be easily tuned to represent a good or bad solvent for the macroions. In this work the *ε* of all pair interactions between all kinds of species is set to 4.5 kJ/mol, and all *σ* is set to 5 Å in order to obtain a good solvent environment. The cut-off distance *r*_*c*_ is set to 15 Å for all LJ interactions.

Furthermore, the interactions between the charged beads on the surface of the macroions and the corresponding counterions in the solution were described by the Coulomb pair-potential


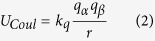


where *U*_*Coul*_ is the Coulomb potential of pairs of beads separated by a distance of *r, q*_*α*_ and *q*_*β*_ are the charges on each bead, and *k*_*q*_ = *1*/*4πε*_0_, where *ε*_0_ is the permittivity of vacuum.

The molecular dynamics (MD) package LAMMPS was used to run all the simulations. The radius of each uncharged bead on the surface of a macroion, as well as each solvent molecule and counterion, was set to 5 Å. Long range Coulombic interactions were calculated using the particle-particle/particle-mesh (PPPM) Ewald algorithm^31^. Each macroion in the present work had a diameter of 25 Å, and all the beads on each macroion move together as a rigid body using LAMMPS inbuilt RIGID package. Every charged bead on macroions’ surfaces was carrying 1 electron charge, while every counterion was carrying 1 elementary positive charge. Moreover, the total number of counterions in the solution was the same as the total number of charged beads on macroions’ surfaces to keep the system electrically neutral. Each simulation began with distributing all the macroions, solvents, and counterions randomly in the solution. All systems were run under isothermal-isobaric (NPT) ensemble. The temperature and pressure of each simulation were set to keep the solvent in a liquid phase and also to enable the solvent molecules to have relatively high mobility. All simulations were run long enough to reach steady states in both static and dynamic properties of the self-assembly process in solution. The time step for all simulations was 10 fs and the systems were equilibrated between 100 to 200 ns depending on the dynamics of the self-assembly process. After equilibration, all systems maintained a stable density around 0.97 ± 0.005 g/cm^3^ along the rest of the simulations.

### Macroions vs. uncharged macromolecules

In this comparison, two systems have been tested: macroions with 20 charged beads on the surface and uncharged macromolecules. There were 27 macroions in the charged system, same as the number of uncharged macromolecules in the uncharged system. Both systems had the same number of solvent molecules, about 200,000. All other conditions were set to the same values for the two systems. After about 500 ns of simulation time, the macroions in both systems have been staying in either aggregated or scattered states for quite a long time, which was confirmed through visualization and radial distribution function (RDF) calculations. Figure S2 shows the RDF, g(r), of the two systems at the end of each simulation, indicating the large difference in the nature of their assembly. The uncharged macromolecules did not show any sign of aggregation, but the small peak manifested in the g(r) could represent short-lived close pairs of uncharged macromolecules.

### Calculating the force between macroions

To accurately confirm the fact that electrostatic interactions are the main reason for the self-assembly of macroions in solution and to quantitatively investigate the nature of the electrostatic force, we have calculated the net VDW and Coulomb forces exerted on each macroion by the surrounding particles for a system which had only two macroions in the solution. The forces then were projected onto the vector pointing from the middle point of the two macroions to the center of each macroion to calculate the contribution from different interactions to the self-assembly. Each of the two macroions in the representative system had 20 charges on the surface surrounded by 40 counterions and about 200,000 solvent molecules, all distributed randomly in the solution at the beginning of the simulation. The VDW and electrostatic forces were both calculated for all species, including the counterions and solvent molecules. Aside from what has been discussed in the main article about Figs 4 and S3 shows the projected VDW forces on one of the two macroions as a function of the distance between them. The value of the resultant VDW projected force is about two-orders of magnitude smaller than the electrostatic forces on the same macroion shown in Fig. S3. The simulation had been repeated more than 5 times by placing the two macroions at different locations in the box at the beginning of each simulation. The two macroions formed a stable dimer pair in all cases, but the time needed to form the dimer varied significantly between different simulations, mainly due to the rapidly changing, non-monotonic electrostatic forces.

### Effect of charge density

In the study of the effect of charge density on the self-assembly process, we have prepared about twenty different macroion solution systems. All systems had the same number of macroions (27), same number of total beads on the surface of the macroions, and same concentration of the solutions (about 200,000 solvent molecules in solution). In each system, the macroions had different number of charges on the surface, from 0 to 68, with various corresponding numbers of counterions to neutralize the negative charges. Simulations of all systems have been run for a minimum of 100 ns to ensure the structures of aggregates were stable. Figure 4a–h shows the snapshots of the final stable aggregated states for eight different macroion systems.

### Dynamics study of macroion systems

The dynamics of macroions, counterions and solvent molecules in all simulations were quantified using time-averaged mean-squared displacement (〈r^2^〉) calculations. The diffusion coefficients were then calculated from linear fits to the mean-squared data and applying the relation *D* = 〈*r*^*2*^〉/*6t*, where D is the diffusion coefficient. For each system the diffusion coefficient was obtained after the aggregated structures were stable for more than 100 ns.

## Additional Information

**How to cite this article**: Liu, Z. *et al*. Elucidating the Origin of the Attractive Force among Hydrophilic Macroions. *Sci. Rep.* **6**, 26595; doi: 10.1038/srep26595 (2016).

## Supplementary Material

Supplementary Information

## Figures and Tables

**Figure 1:**
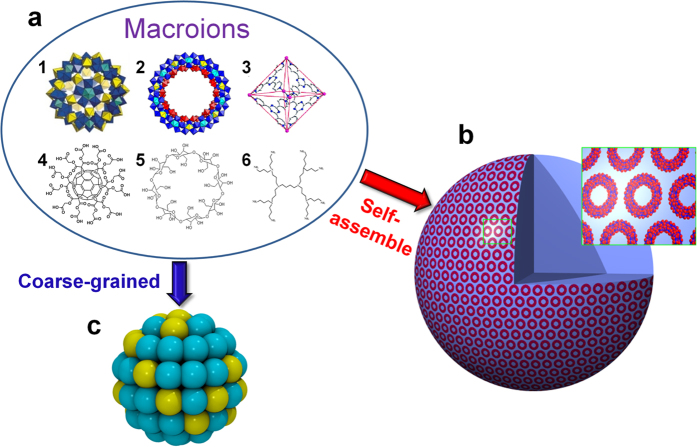
Coarse-graining of various macroions that form blackberries. (**a**) Examples of different kinds of macroions that are able to form blackberries, including inorganic metal-oxide molecular clusters (1, 2)^13^, metal-organic nanocages (3)^32^, functionalized fullerenes (4)^33^, cyclodextrins (5)^34^, dendrimers (6)^35^. (**b**) A typical blackberry structure self-assembled from metal-oxide molecular clusters (a2), which is a highly ordered monolayer hollow sphere. (**c**) A coarse-grained model designed for general spherical macroions. In this model, the cyan beads have only VDW interactions while the yellow beads have both VDW and electrostatic interactions.
