# Supplementary material for: Elucidating the Origin of the Attractive Force among Hydrophilic Macroions
Source: Sci Rep. 2016 May 24;6:26595. doi: 10.1038/srep26595 (PMC4877594; doi:10.1038/srep26595)
Supplement: Supplementary Information [file srep26595-s1.pdf]

Supplementary Information for

Elucidating the Origin of the Attractive Force among Hydrophilic Macroions

Zhuonan Liu, Tianbo Liu*, Mesfin Tsige*

*Corresponding author. E-mail: mtsige@uakron.edu (M.T.) and tliu@uakron.edu (T.L).

This file includes:

Figures S1-S4

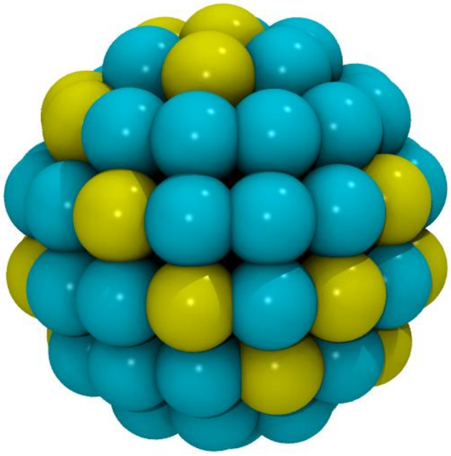

Figure S1. The coarse-grained model for macroions. A hollow spherical model is built to represent various kinds of macroions. The cyan beads have only van der Waals interactions while the yellow beads have both van der Waals and electrostatic interactions. However, there are no intra-molecular interactions between the beads of a macroion and these beads move as one rigid body.

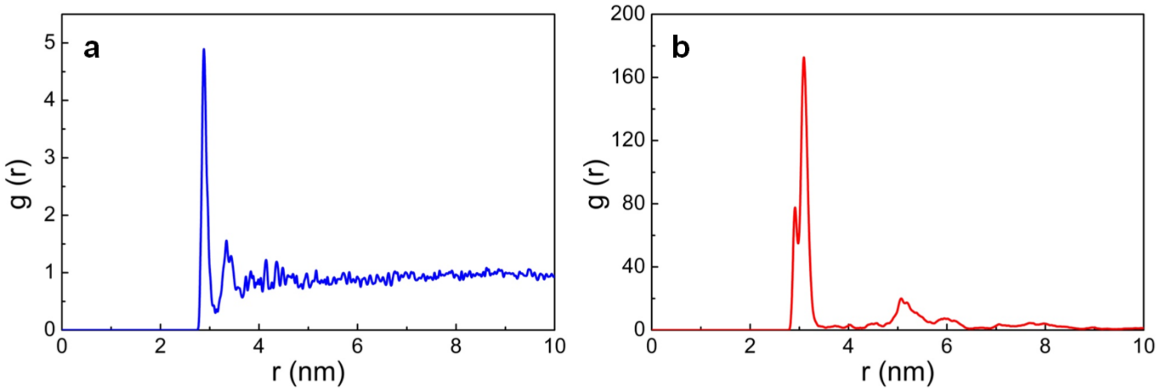

Figure S2. The radial distribution function (RDF) of macroions in solution. (**a**) The RDF of uncharged macromolecules in a solution containing 27 macroions after 500 ns of MD simulation. The first peak (whose amplitude is a small fraction of the amplitude of the first peak shown in (b)) represents an occasional formation of dimers of macroions with a short lifetime, in the order of a few ns. The dimers never had a chance to grow into bigger aggregates because the van der Waals interaction between the macroions was so small that thermal fluctuations could quickly break the dimers into isolated macroions. (**b**) The RDF of the corresponding charged macroion system after 500 ns of MD simulation. The first peak corresponds to the direct contact of macroions, and the second peak implies that the contact is intermediated by one counterion or solvent molecule. The first two high peaks are clear evidences of the existence of the self-assembly, in contrast with the small ones that the uncharged macromolecule system has.

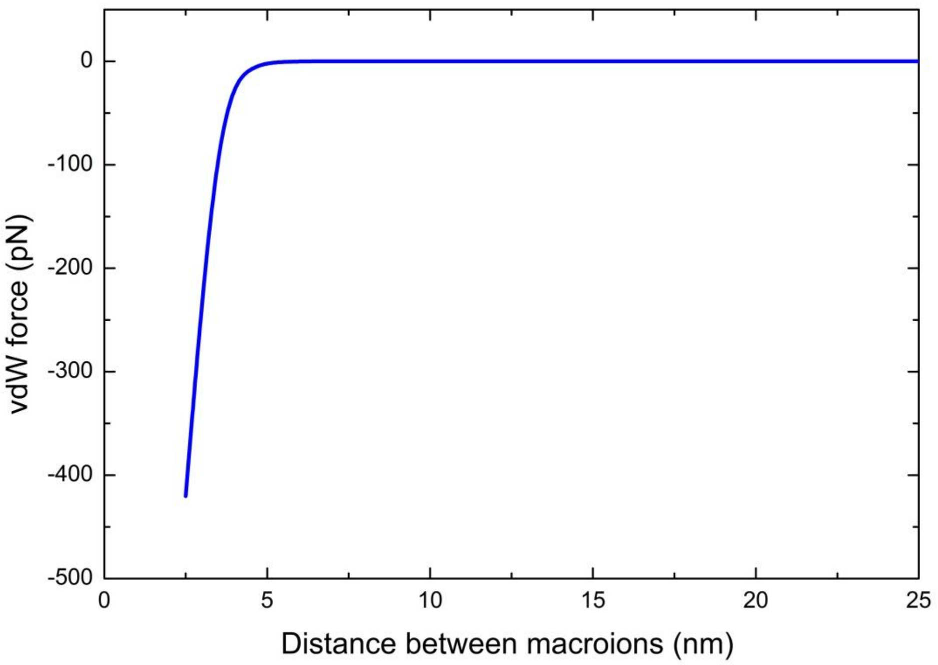

Figure S3. van der Waals interactions between two macroions. The net van der Waals force exerted on one of the two macroions is projected on the vector that points from the midpoint of two macroions to the center of the selected macroion. This calculation is designed for determining the contribution from van der Waals interactions towards the dimer formation. In this calculation a negative value of van der Waals force means an attractive force between the two macroions. The projected van der Waals forces calculated here are about two-orders of magnitude smaller than the projected electrostatic forces (as shown in Fig. 3 in the main article).

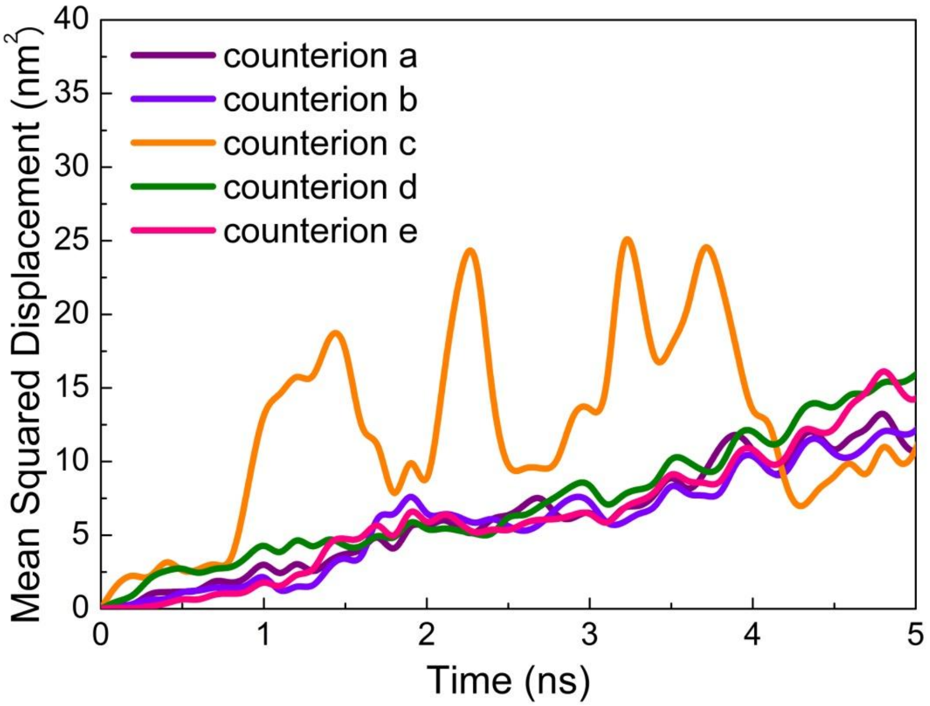

Figure S4. Representative dynamics of counterions. For the case of moderate charge density on macroions (10 charge case), five counterions were randomly picked in the solution and their mean-squared displacements are shown. Most of the counterions show a similar dynamics while "counterion c" displays a completely different dynamics. This counterion was found to associate and disassociate (or freely moving) in the solution multiple times and that behavior was also displayed by several other counterions. Note: in this calculation no time average was involved since the goal is to capture the instant dynamics of counterions.
